# ‘Helpful’, ‘Objective’, and ‘Useful’: User Perceptions of the Animal Welfare Assessment Grid (AWAG) for Dogs as a Decision‐Making Tool

**DOI:** 10.1002/vms3.70993

**Published:** 2026-06-19

**Authors:** Rachel Malkani, Sharmini Julita Paramasivam, Sarah Wolfensohn

**Affiliations:** ^1^ University of Surrey School of Veterinary Medicine Guildford UK

**Keywords:** animal welfare, decision‐making, dog, quality of life, welfare assessment

## Abstract

**Objectives:**

Ethical decision‐making in veterinary practice and in animal welfare sectors can be particularly challenging. Decision‐making is reported to be largely subjective with various influences biasing these decisions. To enhance the objectivity with ethical dilemmas and difficult decisions, the application of standardised tools is increasingly advocated. This study explores the utility of the Animal Welfare Assessment Grid (AWAG) as a decision‐making tool in various sectors of dog welfare including veterinary medicine, shelter environments, and assistance dog organisations.

**Methods:**

This was undertaken through an online mixed‐methods questionnaire of clinicians using the AWAG in their workplace to assess dogs. A total of 38 respondents from a group of 99 veterinary and animal welfare professionals provided insights on the tool's functionality.

**Results:**

Over 96% of respondents acknowledge the utility of the AWAG in aiding in decision‐making and it is reported to facilitate discussions among colleagues and dog caregivers about welfare. While the overall sentiment towards the AWAG was predominantly positive, a minority of users expressed difficulties in using the tool, indicating areas where the tool could be further refined and improved.

**Clinical significance:**

The results suggest that the AWAG serves as a valuable asset for veterinary clinicians and animal welfare professionals, aiding in the assessment of dogs' welfare and informing treatment and management decisions. By providing an instrument to aid in objective reasoning to the complex process of decision‐making, this may help transform how welfare considerations are integrated into daily practice.

## Graphical abstract

Users of the Animal Welfare Assessment Grid (AWAG) found the tool to assist in making decisions and found it enhanced the objectivity of welfare assessment. It also helped facilitate discussions related to the welfare of dogs. The most common words used to describe the tool were useful, objective, and helpful

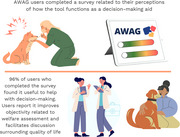

## 1 Introduction

Decision‐making in veterinary medicine and animal welfare has become more complex with new ethical challenges presented. There is now increased public awareness regarding animal welfare in relation to pain and mental wellbeing and growing political pressure to prevent animal suffering. Additionally, the growth in veterinary specialisation and technology allows clinicians to refer patients and undertake complex treatments more readily (Rollin 2006).

Advances in veterinary medicine mean prolongation of life is more easily achievable than in the past, and there is increasing concern over whether this is in the best interests of the animal (Nolff et al. 2016; Sandøe et al. 2016). Treatments may be particularly invasive, extensive, and create unnecessary risk (Yeates 2016). Furthermore, demand for innovative treatments can lead to decision‐making challenges and moral injury in veterinary professionals (Williamson et al. 2023).

In veterinary practice, there is the potential for clinicians to carry out medical and surgical procedures that have been motivated by a) professional advancement, b) financial incentive for the practice, (c) training opportunities, and (d) meeting the somewhat unrealistic expectations of the client (Grimm et al. 2018; Rosoff et al. 2018). These motivations do not necessarily improve the patient's welfare, nor can they be ethically justified.

There are also challenges for rehoming centres and animal welfare charities related to decision‐making. These may include deciding whether to place a dog in a home (Griffin et al. 2020), if a dog should undergo treatment and management interventions, or if staff think a dog will cope in a shelter environment (Cussen and DiGangi 2022). The kennel environment can be challenging for some dogs, and the cumulative stress can have severe welfare implications (Righi et al. 2019). This raises important ethical considerations about the suitability of kennels for long‐term housing in some dogs (Taylor and Mills 2007). Failure to recognise an ethical component of decision‐making can lead to improper decision‐making and unethical outcomes (Moses et al. 2018). Ethical decision‐making can be incredibly difficult due to different interests at stake, and these can often conflict (Vettical 2018). Therefore, tools to improve the decision‐making process are of value in different animal welfare settings.

Tools that can assess both current and future welfare of the dog to assess the benefits and harms of treatment can provide a more objective approach to complex decisions. There are a number of existing validated quality‐of‐life and welfare assessment tools for dogs; however, most are designed to be disease‐specific or are owner‐led. The Animal Welfare Assessment Grid (AWAG) is a validated tool across a range of species that assesses the quality of life (QOL) of an animal over time (Malkani et al. 2022; Ryan et al. 2021; Wolfensohn and Honess 2007). It assesses four parameters (physical health, psychological health, the environment, and any procedural or management events) that reflect the five domains of welfare.

Under each parameter, a range of factors are scored from one (best welfare possible) to ten (worst welfare possible), using mutually exclusive descriptors for each score to reduce bias and subjectivity. The AWAG output produces a cumulative welfare assessment score (CWAS) and mean scores for each parameter. A range of visualisations are produced, allowing clinicians and owners to clearly see how their dog's welfare changes over time, and how it compares with scores of an average healthy dog.

Previous studies using the dog tool have been applied across varying environments to assess welfare and examine with factors are predictive of chronic pain and behavioural disorders in dogs (Malkani et al. 2024). Collectively, these studies highlight that the AWAG can be used to quantify welfare across different conditions and environments, supporting both clinical and behavioural assessments. Its applicability across veterinary practice, shelter environments, and assistance dog settings provides a useful tool for this study.

This study aims to address these concerns by evaluating how the AWAG functions as a decision‐making tool to help with ethical challenges, treatment and management decisions across various dog welfare sectors, including veterinary medicine, shelter environments, and assistance dog organisations, using a mixed‐methods approach. In this study, ‘decision‐making’ refers to clinical and management, and = decisions that directly impact a dog's welfare such as treatment and intervention choices in veterinary practice, quality‐of‐life decisions, and pathway decisions in rehoming and assistance dog organisations (suitability for foster, rehoming, placement, or alternative outcomes).

## 2 Methods

Using the AWAG database (which only the authors RM and SW have access to), veterinary and animal welfare professionals (*n* = 99) that were actively using the AWAG in their workplace across the UK and Australia in veterinary clinics, rehoming shelters, and assistance dog organisations were emailed a questionnaire (Appendix 1) using the University of Surrey Qualtrics XM Platform. All registered users had completed standardised training using AWAG instructional training videos and scoring methodology. Although the training was consistent, participants varied in their length of use. When completing the survey, job role was not captured as this information could potentially identify individuals.

The questionnaire contained two Likert questions related to how they believed the AWAG performed as a decision‐making tool and if the AWAG made them consider factors that they would not normally consider. Following these two questions, respondents were asked to expand on their answer using open‐ended text. Subsequent questions were purely qualitative asking how they used the AWAG to influence dog welfare and how the AWAG influenced their workplace. The final questions asked users to describe the AWAG in three words to capture salient information about users’ perceptions of the tool.

### 2.1 Quantitative Analysis

Quantitative data from Likert‐scale questions were analysed using descriptive statistics only due to the small sample size and the exploratory nature of the study. Therefore, no statistical analyses were undertaken.

### 2.2 Qualitative Analysis

Qualitative data were analysed using thematic and sentiment analysis. The thematic analysis following Braun's six‐step method approach (Braun and Clarke 2006). This involved: (1) familiarisation with the data (2) generating initial themes, highlighting words and phrases relevant to the research questions, (3) generating subthemes, (4) reviewing themes, (5) defining final key themes, and (6) interpreting and reporting. Datasets were analysed using an inductive approach and no predefined categories or themes were used based on the research questions to attempt to reduce bias.

Sentiment analysis was performed using the ‘tm’ package in R Statistical Software (v4.0.1) (R Core Team 2021) to clean the text and remove common stopwords, such as ‘and’, ‘the’, and ‘is’, reducing noise in the analysis. The ‘bing’ lexicon from the ‘tidytext’ package was used to perform sentiment analysis. This lexicon categorises words as either positive or negative based on sentiment. These were then screened manually by the lead author for accuracy and were also further categorised as being mixed or neutral in addition to positive and negative.

## 3 Results

### 3.1 Quantitative Analysis of User Perceptions of How the Tool Contributed to Overall Treatment and Management Decisions

Thirty‐eight AWAG users completed the online survey. When AWAG users were asked how the tool contributed to overall treatment and management decisions of the dog, 22 users found the tool to be very useful or extremely useful, and 12 found it to be moderately useful. Two users found it to be slightly useful, and one not at all useful.

### 3.2 Qualitative Analysis of User Perceptions of How the Tool Contributed to Overall Treatment and Management Decisions

Thematic analysis revealed three main themes related to decision‐making using the AWAG. The main themes identified were objectivity and science‐based, change and approach, and benefits, utility, and challenges (Table 1). Sentiment analysis was also undertaken on the qualitative data in this question. Twenty‐three of the responses were deemed to be positive, two were categorised as very positive, and four were negative.

**TABLE 1:** Quotes given by AWAG users when asked how the tool contributed to the overall treatment and management decisions of the dog categorised under three themes.

AWAG quotations
**Objective and science‐based**
‘It provides an objective approach to assessing an animal and helps to keep track of any progress and decline as well as what is contributing to the dog's behaviour’.
‘It has provided a science‐based approach to recording deterioration and improvement to the overall condition of the animal. This is helpful when determining the best possible pathways for those in care’.
‘It gives valuable objective information and provides a clear indication of changes in subsequent assessments’.
‘I found that the AWAG provided a black and white assessment for the dog which helped with decision‐making and assessing his quality of life’.
**Change and approach**
‘By understanding whether it was procedural/environment etc gave us the tools to be able to focus on the area to improve’.
‘Highlighted areas that required intervention. If changes were made and still presented high scores/welfare compromised, assisted in determining pathway. Translated observations and discussion into data, which provided another tool when communicating outcomes/pathways’.
**Benefits, utility, and challenges**
‘By understanding whether it was procedural/environment etc gave us the tools to be able to focus on the area to improve’.
‘Useful to demonstrate subtle changes to the caregivers and also have something to send over to the referring vets’.
‘We were limited in its use due to the time‐consuming nature of the questionnaire, but when we could use it, we were able to change our approach to the patient with a positive outcome’.

Users of the AWAG were asked ‘did the AWAG encourage you to consider factors/a factor (behaviour, environment, procedural and management events, etc.) that you wouldn't normally when assessing welfare?’. Twenty‐six respondents answered yes and 12 answered no. Qualitative analysis found two main themes were expressed in the responses to this question pertaining to the approach to welfare assessment and how the AWAG influenced management and decision‐making. Sentiment analysis revealed that the responses were largely positive (*n* = 22) or mixed (*n* = 11) with no negative sentiment. AWAG users also report that the tool helps to identify where welfare may be poor and interventions need to be made, or it aids in discerning when a particular approach may not be benefiting the dog in question (Table 2).

**TABLE 2:** Quotes from AWAG users discussing how the AWAG encouraged them to consider factors that they would not normally when assessing welfare divided into two themes.

AWAG quotations
**Approach to welfare assessment**
‘It helps to provide focus on what is impacting the animal the most and therefore it helps with discussions re how we can change the way we work with the animal’.
‘The factor of procedures and the level of impact which they may/would have long term on the dog and how that may compromise or improve upon their welfare, assisted in decision evidence support’.
‘It opened up discussions on environmental enrichment especially in older dogs with osteoarthritis where walks and play opportunities had diminished’.
**Influenced management and decision‐making**
‘It helps as a measure to trend scores and present to people who aren't regularly working with the case to support decision making’.
‘It helped with some decisions, however mostly helped as a tool for long term animals that were required to stay in custody. It also assisted with a seizure to help look at welfare on the property and also once seized while in care or in foster for the case’.

### 3.3 Qualitative Analysis for How Was the AWAG Used to Influence Welfare

When asked how the AWAG was used to influence welfare, three general themes were identified. These were providing evidence of change, monitoring, and provoking discussions. Regarding providing evidence of change, users of the AWAG indicated that it helped in investigating what was impacting welfare. The tool was reported to demonstrate deterioration in welfare state over‐time and is highlighted to be useful for caregivers who were struggling with decisions about quality of life (Table 3). Sentiment analysis showed that 33 the responses were positive or very positive. Three of the answers were negative and two were shown to be of neutral sentiment. Negative responses were surrounding the scoring, impressions of subjectivity, and usefulness of the AWAG.

**TABLE 3:** Quotes from AWAG users related to how they use the tool to influence welfare categorised into three themes.

AWAG quotations	
**Evidence of change**	
‘We use it whenever we suspect a compromise in welfare but difficult to pinpoint the causes’.	
‘I would use the AWAG tool as a means to demonstrate the “profile” of a dogs wellbeing and how that was changing over time. The visual representation in the graphs was easier for those without welfare knowledge to quantify and understand’.	
**Monitoring**	
‘Mostly to demonstrate deteriorations over time for caregivers who were having a hard time deciding on QOL’.	
‘It has been a tool to officially document changes to the animal's welfare while in shelter and foster care’.	
‘It was used to monitor dogs held in a rescue shelter, either held under statutory powers, or surrendered to decide on appropriate pathways or outcomes and to monitor their behaviour’.	
**Provoking discussions**	
‘Conducting a weekly welfare check and assessing where the progress and decline. Depending on the result it assists with team discussions as well as updates to the management plan or in creating one’.	
‘To open discussions with clients on weight, enrichment activities and potential sources of stress in particular’.	

### 3.4 Qualitative Analysis for How Has the AWAG Impacted Workplaces

The sentiment about how the AWAG has impacted workplaces was generally positive and very positive, which combined were 32 responses. AWAG users were asked to describe how the instrument has impacted their workplace. The main theme identified was behaviour change with subthemes surrounding communication and complex cases (Table 4). Another subtheme is raising awareness of the multiple dimensions of welfare. Neutral answers were about frequency of use in the workplace.

**TABLE 4:** Quotes related to how the AWAG has impacted workplaces split into three themes.

AWAG quotations
**Behaviour change—communication**
‘Allowed better communication and reduction of conflict with case management’.
‘Assists with communicating why decisions are made, collates data and presents it in a different form that some may find easier to interpret/understand’.
**Behaviour change—complex cases**
‘It has assisted with providing evidence of an animals wellbeing at times when difficult euthanasia decisions arise’.
**Multidimensions of welfare**
‘It has prompted the development of an in‐house emotional welfare protocol to help encourage a holistic view of our patients’.
‘Positively. It has also been really instrumental in getting people to think of the dog in a holistic way and also raised the profile of dog wellbeing and the interplay between health and behaviour’.

### 3.5 Words Used to Describe the AWAG

Respondents were asked to describe the AWAG in three words. Core themes were identified which included utility & functionality. Words such as ‘helpful’, ‘useful’, ‘amazing’, ‘beneficial’, ‘essential’, and ‘impactful’ suggest that the AWAG is perceived as a valuable and practical tool. Descriptions such as ‘measuring tool’, ‘assessment tool’, ‘tracking tool’, ‘monitoring tool’, and ‘decision‐making tool’ further highlight its functional use. Visual representation was another core theme derived in the analysis. The descriptors ‘visual’ and ‘visually’ indicate that the AWAG provides a graphical representation of welfare. The theme of usability was also identified; use of words such as ‘easy’, ‘effective’, and ‘clear’ point to ease of use. However, the term ‘complicated’ (which was used by one respondent) suggests that some users may have found certain aspects of the tool challenging. Emotional descriptions were another main theme through use of the words ‘ground‐breaking’, ‘innovative’, ‘progressive’, ‘provoking’, and ‘interesting’. A final theme was focus on welfare. The recurring mention of welfare in various responses such as ‘welfare tool’, ‘welfare tracking’, and ‘welfare assessment’ reflects the tool's primary purpose and focus. The most common words were ‘helpful’, ‘objective’, and ‘useful’ (Table 5).

**TABLE 5:** Most common word used words to describe the AWAG, themes, and sentiment.

Theme	Words used	Frequency	Sentiment
Utility & functionality	helpful, useful, beneficial, essential, effective	16	Positive
Innovation	innovative, progressive, interesting, impactful	13	Positive
Objectivity & measurement	objective, measuring, data, assessment	12	Positive
Visual representation	visual, monitoring, tracking	11	Positive
Usability	easy, clear	7	Positive
Challenges	complicated	1	Negative

## 4 Discussion

The findings of this study suggest that the Animal Welfare Assessment Grid (AWAG) is largely perceived by users as a useful framework to support treatment and management decisions in canine welfare contexts. Respondents highlighted its structured and multidimensional approach, and described the tool as contributing to greater clarity in decision‐making processes, especially in complex or longitudinal cases. While welfare assessment remains inherently subjective, users reported that the AWAG provided a consistent framework that helped to structure professional reasoning and prioritise interventions.

The AWAG has been assessed for construct validity alongside clinical measures in previous studies, including chronic disease and short‐term procedural changes in welfare (neutering) to ensure it detects changes in welfare state (Malkani et al. 2022). However, establishing concurrent criterion validity for welfare assessment tools remains inherently challenging, as no single gold standard measure of animal welfare currently exists. As a result, the AWAG is best considered a complementary holistic framework, rather than a replacement for condition‐specific measures.

It is a promising finding that over 70% of users found that the AWAG encouraged them to consider aspects of welfare that may normally be overlooked during their assessments. As veterinary surgeons are shown to have a focus on physical health (Koch 2009; Serpell 2019), using a tool such as the AWAG facilitates the consideration of factors that are determinants of dog welfare that might not have been previously acknowledged, thereby potentially preventing significant welfare improvements. Qualitative feedback from users indicates that the AWAG may have broadened their view of welfare evaluation, encouraging the inclusion of previously overlooked factors such as the environment.

A promising theme discerned was how the AWAG prompted discussions about welfare; it is reported to facilitate meaningful and often difficult discussions among staff and clients. Additionally, visual outputs of the AWAG can highlight areas of concern or improvement, making it easier for individuals without specialised knowledge to understand and engage in welfare discussions, which may enable them to recognise and prioritise the welfare of dogs in their care. The AWAG has also facilitated behaviour change by encouraging staff to think about welfare in a more holistic manner. Having the ability to quantify welfare and present data comprehensively, can made interpretations and understandings of quality of life more accessible, particularly in challenging situations such as euthanasia discussions.

Tools to facilitate shared decision‐making in human medicine have been shown to improve the decision‐making process and allows patients to evaluate and visualise potential outcomes of various treatments (Braillon and Bewley 2015; Hofmann et al. 2012; Saposnik et al. 2016). Human patients increasingly desire to participate actively in their healthcare planning and to be fully informed about their treatment options (Hofmann et al. 2012). Similarly, this desire for engagement and informed decision‐making extends to dog owners regarding their pets' medical care. Correspondingly, just as various tools in human medicine facilitate shared decision‐making, the AWAG has shown to be similarly advantageous. It provides a platform to collaborate effectively, ensuring that decisions made are based on animal welfare rather than owner expectations.

Sentiment analysis of the qualitative responses across all questions demonstrates the predominantly positive perception of the AWAG, with the majority of data classified as positive. Nonetheless, the presence of negative sentiment and neutral, albeit minimal, is a critical reminder that there is room for improvement. Neutral sentiment may reflect users' uncertainty or lack of familiarity with the AWAG's use and capabilities. Some respondents indicated infrequent use as reasons for neutral feedback, suggesting that while they recognise the AWAG's potential utility, they have not fully integrated it into their welfare assessments. This sentiment could point to a need for greater training and support to improve understanding and use of the tool. It might also suggest a hesitancy to rely on tools or a belief they are unnecessary, reflecting a traditional approach in veterinary practice that prioritises health concerns. The focus on physical health concerns might be overshadowing the importance of other welfare factors, which are integral to clinical decision‐making.

Overall, it appears that the AWAG has been a useful tool for clinicians and animal welfare professionals to assess the welfare of dogs and to use these parameters to help make treatment and management decisions. In the context of ethical decision‐making, the integration of the Veterinary Ethics Tool (Grimm et al. 2018) with the AWAG offers a robust framework for addressing ethical dilemmas. This combined approach allows for a more nuanced consideration of both ethical and welfare aspects in treatment decision‐making. It encourages discussions that are not only informed by the physical and psychological state of the animal, as quantified by AWAG, but also by the ethical implications of potential interventions. Such discussions ensure that the decisions made are not only in the best interest of the animal, but also align with good ethical veterinary practice (Hernandez et al. 2018).

Moreover, this integrated approach can be particularly valuable in complex cases where treatment decisions might involve significant trade‐offs or potential conflicts between what is medically feasible and what is ethically appropriate. It allows for a balanced consideration of the animal's quality of life, the owner's perspectives, and the veterinary surgeon's professional judgment. This holistic approach, combining AWAG's detailed welfare assessment with ethical considerations, may advance the field of veterinary medicine for more comprehensive and compassionate veterinary care. It highlights the importance of considering the multidimensional aspect of welfare, not just at the time of assessment, but also in the context of their longer‐term quality of life, which may make the CWAS (the output score generated by the AWAG algorithm) an important part of longitudinal monitoring.

## 5 Limitations

The AWAG and the study methodology are not without limitations. Respondents represented veterinary settings and animal charities; however, the analysis did not differentiate between these demographics due to insufficient sample size, leaving unanswered questions regarding the potential variance in the AWAG's value across these environments. Moreover, job roles and length of use were not captured. This limitation indicates a need for future research to determine if the tool's utility is influenced by the context in which it is applied. Furthermore, it is important to note that the sample of users is likely biased towards individuals who were not only inclined to use the AWAG but also willing to complete this questionnaire. This suggests that these users were probably already invested in improving welfare, which could have influenced their perspectives and responses.

The AWAG for dog's generic design may limit sensitivity to issues specific to particular subpopulations or environments. Dogs in specialised contexts such as those involved in complex behavioural rehabilitation may experience welfare challenges that are not fully captured within the tool. To mitigate this, the AWAG allows for the inclusion of additional where appropriate; these require testing to ensure reliability and validity of the tool. Furthermore, although all respondents in this study received standardised training through the AWAG instructional materials, variation in duration and experience of use may have influenced how confidently or consistently the tool was applied.

## 6 Conclusions

This study demonstrates that the AWAG was perceived by users as a useful tool for supporting treatment and management decisions in dogs across veterinary and welfare settings. Most respondents reported that the tool helped them consider welfare factors that might otherwise have been overlooked, helped guide interventions, and facilitated discussions among staff and with clients. The results report that AWAG can help validate choices to those with opposing views, ensuring that the decision‐making process is transparent, inclusive, and evidence based. This can be instrumental in mitigating potential conflict, creating a collaborative environment for addressing animal welfare issues.

## Author Contributions

**Rachel Malkani**: conceptualisation, data curation, formal analysis, investigation, methodology, project administration, software, visualisation, writing – original draft, writing – review & editing. **Sharmini Julita Paramasivam**: supervision, writing – review & editing. **Sarah Wolfensohn**: supervision, writing – review & editing.

## Funding

This research was funded by Agria SKK.

## Conflicts of Interest

The authors declare no conflicts of interest.

## Supporting information




**Supporting Information 1**: vms370993‐sup‐0001‐SuppMat.docx

**Supporting Information 2**: vms370993‐sup‐0002‐SuppMat.xlsx

## Data Availability

All anonymised data are available upon request.
