# Supplementary material for: ‘Helpful’, ‘Objective’, and ‘Useful’: User Perceptions of the Animal Welfare Assessment Grid (AWAG) for Dogs as a Decision‐Making Tool
Source: Vet Med Sci. 2026 Jun 19;12(4):e70993. doi: 10.1002/vms3.70993 (PMC13281402; doi:10.1002/vms3.70993)
Supplement: Supplementary file 1 — Supporting Information 1: vms370993‐sup‐0001‐SuppMat.docx [file VMS3-12-e70993-s002.docx]

Q1 How did the AWAG contribute to the overall treatment and management decisions of the dog?

|  | Not at all useful (7) | Slightly useful (8) | Moderately useful (9) | Very useful (10) | Extremely useful (11) |
| --- | --- | --- | --- | --- | --- |
| Select one (7) |  |  |  |  |  |

Q1a Please expand here

________________________________________________________________

| Page Break |  |
| --- | --- |

Q2 Did the AWAG encourage you to consider factors / a factor (behaviour, environment, procedural and management events etc) that you wouldn’t normally when assessing welfare?

|  | No (1) | Yes (2) |
| --- | --- | --- |
| Select one (1) |  |  |

Q2a Please expand here

________________________________________________________________

| Page Break |  |
| --- | --- |

Q3 How did you use the AWAG to influence dog welfare

________________________________________________________________

| Page Break |  |
| --- | --- |

Q4 Please describe how the AWAG has impacted your workplace

________________________________________________________________

| Page Break |  |
| --- | --- |

Q5 Please describe the AWAG in three words

________________________________________________________________

End of Block: Default Question Block
